#  Neurologic Manifestations of Childhood Rheumatic Diseases

**Published:** 2012

**Authors:** Reza SHIARI

**Affiliations:** Associate Professor of Pediatric Rheumatology, Department of Pediatric Rheumatology, Faculty of Medicine, Shahid Beheshti University of Medical Sciences, Tehran- Iran

**Keywords:** Neurologic manifestations, Systemic Lupus erythematosus, Kawasaki disease, Seizures, Aseptic meningitis

## Abstract

Children with rheumatic disorders may have a wide variety of clinical features ranging from fever or a simple arthritis to complex multisystem autoimmune diseases. Information about the prevalence of neurological manifestations in children with rheumatologic disorders is limited. This review describes the neurologic complications of childhood Rheumatic disease either solely or combined with symptoms of other organs involvement, as a primary manifestation or as a part of other symptoms, additionally.

## Introduction

Neurologic manifestations of rheumatic disorders occur in two forms of primary and secondary (1). In primary central nervous system (CNS) involvement, inflammation is limited to the brain and spinal cord. However, secondary CNS involvement occurs in a variety of conditions including infections, malignancies, systemic vasculitides and other collagen vascular diseases (2). Herein, we have reviewed the common rheumatic diseases of childhood and their neurological manifestations and complications in the central and peripheral nervous system as a primary manifestation or as a part of other symptoms, as well.

**Henoch-Schönlein Purpura**

Henoch-Schönlein purpura (HSP) is the most common vasculitis of small vessels in childhood. Approximately 75% of the cases occur in children aged 2-11 years. The median age is 5 years (3). HSP is a multisystem disease that affects the skin, joints, gastrointestinal tract and kidneys; however, other organs such as the neural system may be affected. The etiology of HSP is not fully clear; nonetheless, the genetics, environment and antigenic components are involved in it. An antecedent upper-respiratory tract infection has been discovered in most children. Bacterial and viral infectious agents, drug ingestions and vaccinations have been associated with the development of Henoch-Schönlein purpura (4).

Ostergaard et al. (5) reported the neurological manifestations of 26 children with HSP. They did not find any cases with seizures, aphasia, ataxia, paresis or cerebral hemorrhage, but behavioral changes were present in eight patients (31%). EEGabnormalities like slow wave foci, sharp waves and paroxysms were demonstrated in 12 patients (46%) during the acute phase of the disease as well. One year followup of these patients showed that only in four patients the EEG-changes were persistent. There was a significant association between the presence of headache and EEG abnormalities. Although the long-term prognosis of HSP is almost individually ascribed to renal disease, some rare extra-renal complications may cause morbidity. For this concern, there are several case reports that show cerebral vasculitis in children caused by Henoch-Schonlein purpura (6-8).

**Kawasaki Disease**

Kawasaki disease (KD) also known as mucocutaneous lymph node syndrome is an acute febrile multisystem vasculitis affecting medium sized vessels. It usually affects children under five years of age and is associated with conjunctival and oral erythema, edema of the palms and soles (9). Coronary artery aneurysms occur in 25% of untreated cases, which may result in myocardial infarction. It is evident that the possibility of atypical KD should be considered whenever a child presents with prolonged fever and myocarditis in association with a very high level of ESR (10). Aseptic meningitis is the most common neurologic manifestation of KD; however, hemiplegic strokes, acute encephalopathy, irritability and facial palsy are also described (11, 12). In children with KD, there is an acute systemic inflammatory vasculitis without fibrinoid necrosis. There is also a possible role for anti-endothelial cell antibodies in the pathogenesis of the disease (13, 14). 

**Systemic Lupus Erythematosus**

Systemic lupus erythematosus (SLE) is an autoimmune and multi-system disease with a various spectrum of clinical and immune abnormalities. Despite advances in our understanding, the etiology of SLE remains unknown (15, 16). Estimates of the prevalence of neuropsychiatric systemic lupus erythematosus (NPSLE) have ranged from 22% to 95% in children (17, 18). Central nervous system (CNS) lupus is a severe disease with diagnostic challenges. SLE is sometimes diagnosed after patients present for treatment of a neurologic event and numerous children with SLE have neurologic symptoms of the disease (19, 20). Neuropsychiatric syndromes associated with SLE are aseptic meningitis, stroke, demyelinating syndrome, headache, chorea, myelopathy, seizures, anxiety/ mood disorders, psychosis, Guillain-Barre´ syndrome, plexopathy, cranial and/or peripheral neuropathy, myasthenia gravis, autonomic disorder and migraine (20, 21).

Mild to moderate mental retardation and serious emotional and social problems might be the first manifestation of children with SLE who are complicated with Klinefelter’s syndrome (22). Drug induced lupus (eg, phenytoin, hydralazine, procainamide and isoniazid) is different from classic SLE. Usually, in drug induced lupus, antihistone antibody is positive and there is neither kidney nor CNS involvement (23). 

Autoantibody against the N-terminal 120 kDa form of a-fodrin is a specific and sensitive disease marker for both juvenile and adult Sjogren syndrome. Epitope mapping of this protein using dot blot analysis have shown that a-fodrin is in association with the development of neurological complications or the disease progression of both Sjogren and SLE (24). 

**Behcet’s Disease**

Behcet’s disease (BD) is a multisystem disease with an unknown etiology, characterized by recurrent oral and genitalia ulceration and panuveitis. Recent studies have shown that BD is much more frequent than we had thought and genetic factors have long been implicated in the disease (25). Neurologic involvement in Behçet disease was first reported by Knapp in 1941. Pallis and Fudge (1956) and Wadia and Williams (1957) described the clinical syndromes and classification of BD later (26).

Neuro-Behçet’s disease (NBD) is caused by two mechanisms; which are as following; 1. primary neural parenchymal lesions, 2.secondary to major vascular involvement (27). These two types of involvement rarely occur in the same individual. Meningoencephalitis, spinal cord syndromes, meningitis and encephalopathy, optic neuropathy, facial weakness, vestibular disturbance, vertigo, sensory neural deafness, cortical venous thrombosis, intercranial hypertension, ataxia, ocular motor dysfunction, dysarthria, dysphagia, isolated cranial nerve lesions (nerve VII was the most), severe transverse myelitis with paraplegia, Brown Sequard syndrome, hemiparesis and hemisensory disturbance, seizure and hippocampal complex partial seizures have been reported (27-31). 

Ideguchi et al. studied 412 patients with BD, of whom 54 (13%) had neuro-Behçet’s disease (NBD). According to their report, NBD was significantly higher in males (P = 0.009). The majority of patients (n = 38, 70%) had acute parenchymal NBD, 15 (28%) had chronic progressive parenchymal NBD and one (2%) had the non-parenchymal type. Headache and fever were more frequently reported by patients with acute parenchymal involvement. Personality changes, sphincter disturbances, involuntary movements and ataxia occurred predominantly in patients with chronic progressive parenchymal disease. Lesions were distributed throughout the CNS, but mainly in the brainstem, white matter and basal ganglia. Analysis of end-point clinical outcomes of their patients revealed a poor prognosis for patients with chronic progressive NBD (32).

**Juvenile Idiopathic Arthritis**

Juvenile idiopathic arthritis (JIA) is the most common pediatric rheumatic disease and is associated with significant long-term morbidity and mortality. JIA is an exclusion diagnosis that applies to any arthritis of unknown persisting for more than 6 weeks with an onset before the age of 16 years (33, 35). A wide spectrum of neurological conditions occur in JIA, mostly in systemic onset JIA including neuropathy, encephalopathy, vasculitis, seizure and macrophage activation syndrome (36-38). Cervical spine disease in polyarthritis JIA can cause atlantoaxial subluxation, which can lead to cord compression, or more commonly, the persistence of a neurological deficit from the spinal cord, with sensory and/or motor signs that usually deserves surgical intervention (39).

**Periodic Fever Syndromes**

Neonatal onset multi-inflammatory disease or chronic infantile neurologic cutaneous and articular (NOMID/CINCA), familial Mediterranean fever (FMF), periodic fever, aphthous stomatitis, pharyngitis, adenitis syndrome (PFAPA) and hyper IgD syndrome are the most common periodic fever syndromes in childhood. NOMID/CINCA syndrome is an unusual disorder that mimics systemic juvenile idiopathic arthritis. Its onset is during the first year of life. Clinical manifestations include hectic fever, intermittent rash, lymphadenopathy, hepatosplenomegaly, uveitis, cognitive and developmental delay, chronic meningitis, hydrocephalus, seizures, papilledema and deforming arthropathy with periosteal changes and bony overgrowth (40). Almost all patients have chronic meningitis from infancy and hydrocephalus, and ventriculomegaly can present in utero. 

Familial Mediterranean fever is an autosomal recessive disorder characterized by recurrent attacks of fever and painful inflammatory manifestations in one or two sites. It is an ethnically restricted genetic disease found commonly among the Mediterranean population, as well as Armenians, Turks, Arabs and Jews. The disease is caused by mutations in the MEFV gene, encoding the Pyrin protein (41). Kalyoncu et al. reported a case series of FMF patients with CNS involvement. According to their study, of the 8864 patients in the genetic testing database, 18 with neurologic signs were assessed. The mean age of the patients was 31.0±11.8 years, the mean age of the first FMF symptom was 12.6±5.6 years, and the mean age of neurologic involvement was 25.8± 12.2 years. Almost 50% of the patients were women. A homozygote MEFV mutation was detected in 16 of 18 patients (88.8%) and a homozygote M694V mutation was found in 72.2% of the patients. They found seven FMF patients with demyelinating lesions, seven with cerebrovascular disease and four with posterior reversible leukoencephalopathy syndrome (PRES). All patients in the PRES group had hypertension.

Demyelinating lesions and cerebrovascular disease were the most common clinical presentations (42). Anti-Neutrophil Cytoplasmic Antibody (ANCA) 

**Associated Vasculitis**

ANCA associated vasculitis (AAV) comprises three syndromes with systemic vasculitis including Wegener’s granulomatosis, Churg Strauss syndrome and microscopic polyangiitis, which all involve small and medium sized vessels and are associated with ANCA (43).

Granulomatosis with polyangitis or Wegener’s granulomatosis (WG) is a vasculitis associated primarily with pulmonary and renal involvement. It is histologically distinguished by the presence of necrosis, granulomatous inflammation and vasculitis. In a large series of 324 patients with WG, 33.6% had neurological involvement. The commonest features included peripheral neuropathies, cranial neuropathy, ophthalmoplegia, cerebrovascular events and seizures (44).

Churg-Strauss syndrome (CSS) or allergic granulomatous angiitis, is a rare syndrome that affects small- to medium-sized arteries and veins. 

The American College of Rheumatology has proposed 6 criteria for the diagnosis of Churg-Strauss syndrome and mononeuritis multiplex or polyneuropathy is one of these criteria (45). In a study conducted by Sehgal et al., of the 47 patients with CSS, 29 (62%) had neurologic involvement. Peripheral neuropathy was detected in 25 patients; of which 17 had multiple mononeuropathy, seven had distal symmetric polyneuropathy and one had asymmetric polyneuropathy (46). Chorea and hemiplegia have also been reported in children with CSS (47, 48).

Polyarteritis nodosa (PAN) is a systemic necrotizing vasculitis that typically affects medium-sized muscular arteries with occasional involvement of small muscular arteries. Microscopic polyangiitis (MPA) has been accepted as individual disease and the usual availability and identification of ANCA testing as important to diagnosing vasculitides other than PAN, have recognized more patients with vasculitis who don’t have simple PAN (49). The neurological conditions found include headache, seizure, stroke, mononeuritis multiplex and symmetrical polyneuropathy (49, 50).

**Primary CNS vasculitis**

Primary CNS vasculitis of childhood is a serious, but potentially reversible inflammatory brain disease (51). 

A small number of case reports have described fatalities resulting from this disease. Although the neurological compromise observed at initial presentation may be very grave and even life-threatening; however, most children survive (52). The Calabrese diagnostic criteria for primary angiitis of the CNS in adults were described in 1992 (53) that were a newly acquired neurological deficit, angiographic and/or histologic evidence of CNS vasculitis and absence of a systemic condition associated with these findings. However, no diagnostic criteria for children are available. Therefore, the adult definition is applied in practice for children. No peak in age distribution has been identified for primary CNS vasculitis. A wide spectrum of clinical presentation in patients with primary CNS vasculitis of childhood is perceived, ranging from an insidious onset of headache, behavior changes or psychosis, cognitive decline, seizures, status epilepticus, cranial nerve palsy, stroke and optic neuritis (mostly bilateral) have also been reported. The presentation may be affected by the type of vascular lesion (large-medium versus small vessel), in addition to the location of detached lesions (54-56).

## Discussion

We have reviewed the CNS manifestations of common rheumatic disorders of childhood including Henoch-Schonlein purpura, Kawasaki disease, systemic lupus erythematosus, Behcet’s disease, juvenile idiopathic arthritis, CINCA syndrome, familial Mediterranean fever, Wegener’s granulomatosis, Churg strauss syndrome, polyarteritis nodosa, and primary CNS vasculitis. The presence and the degree of nervous system impairment vary widely, depending on the diagnosis and course of the disorder. Severe CNS involvement is associated with poor prognosis and high mortality rate. High-dose steroid and cyclophosphamide (oral or intravenous) are first choice drugs in the treatment; plasmapheresis, IVIG, thalidomide and intrathecal treatment may be valuable in treatment-resistant and serious cases. Manifestations of neurologic disease may precede the onset of any other symptoms or may occur much later.
